# *TP53* Arg72Pro polymorphism and neuroblastoma susceptibility in eastern Chinese children: a three-center case–control study

**DOI:** 10.1042/BSR20200854

**Published:** 2020-05-22

**Authors:** Yuan Fang, Xuemei Wu, Lin Li, Jinhong Zhu, Haiyan Wu, Haixia Zhou, Jing He, Yizhen Wang

**Affiliations:** 1 Department of Pathology, Anhui Provincial Children’s Hospital, Hefei 230051, Anhui, China; 2 Clinical Laboratory, Anhui Provincial Children’s Hospital, Hefei 230051, Anhui, China; 3 Department of Clinical Laboratory, Biobank, Harbin Medical University Cancer Hospital, Harbin 150040, Heilongjiang, China; 4 Department of Pathology, Children’s Hospital of Nanjing Medical University, Nanjing 210008, Jiangsu, China; 5 Department of Hematology, The Second Affiliated Hospital and Yuying Children’s Hospital of Wenzhou Medical University, Wenzhou 325027, Zhejiang, China; 6 Department of Pediatric Surgery, Guangzhou Institute of Pediatrics, Guangdong Provincial Key Laboratory of Research in Structural Birth Defect Disease, Guangzhou Women and Children’s Medical Center, Guangzhou Medical University, Guangzhou 510623, Guangdong, China

**Keywords:** Arg72Pro, neuroblastoma, polymorphism, TP53

## Abstract

*TP53* is a tumor suppressor gene that regulates cell growth, apoptosis and DNA repair. Previous studies have reported the contribution of *TP53* Arg72Pro (rs1042522 C>G) polymorphism to pathogenesis of multiple tumors. Hence, we evaluated the association between this polymorphism and neuroblastoma susceptibility in eastern Chinese children. The Taqman genotyping assay was performed in 373 patients and 762 controls. Odds ratios (ORs) with 95% confidence intervals (CIs) were calculated to assess the strength of the association. No significant association was found between the *TP53* gene rs1042522 C>G polymorphism and neuroblastoma susceptibility in the overall analysis (CG vs. CC: adjusted OR = 0.92, 95% CI = 0.70–1.22, *P*=0.567; GG vs. CC: adjusted OR = 0.99, 95% CI = 0.69–1.42, *P*=0.947; CG/GG vs. CC: adjusted OR = 0.94, 95% CI = 0.72–1.23, *P*=0.639; or GG vs. CC/CG: adjusted OR = 1.04, 95% CI = 0.75–1.43, *P*=0.814) and stratified analysis by age, gender, sites of origin, and clinical stages. The *TP53* gene rs1042522 C>G polymorphism may not be a risk factor for neuroblastoma in eastern Chinese children. Future studies are needed to confirm this negative result and to reveal additional functional *TP53* variants predisposing to neuroblastoma.

## Introduction

Neuroblastoma is the most common extracranial malignant solid tumor in children, affecting 25–50 individuals per million worldwide [1], while approximately 7.7 per million in Chinese subjects [2]. Neuroblastoma accounts for 8–10% of childhood malignancies and 10% of tumor deaths [3]. It is an embryonic tumor originating from the primitive neural crest. Lesions are frequently observed in the adrenal gland, retroperitoneum, mediastinum or any spaces flanking the paraspinal sympatheti ganglia. Neuroblastoma is phenotypically hetergeneous. Patients with low- and intermediate-risk neuroblastoma, approximately 60% of cases, benefit from modern multimodality treatments and achieve greater than 95% overall survival [4]. In contrast, only 50% children with high-risk disease can attain long-time survival [4]. Neuroblastoma is also a genetic complex disease. Various genomic features including somatic genetic aberrations, chromosome copy number, transcriptomics, and epigenetics have all implicated in neuroblastoma pathogenesis [1]. However, regarding neuroblastoma risk, no defined environmental risk factors, not even maternal exposures to some harmful materials, have been established, to date. With the advances in the technology, such as genome-wide association study (GWAS), it has been unveiled that germline variations often predispose children to sporadic neuroblastoma, including single-nucleotide polymorphisms (SNPs) in *CASC15, BARD1, LMO1, HACE1, LIN28B, MLF1*, and *CPZ* genes [5–9].

p53 is an important tumor suppressor. Once activated, p53 initiates DNA repair or cell death depending on the degree of damage to maintain genome integrity and stability [10]. Because of its functional importance, *TP53* gene mutations and functional SNPs that affect its tumor suppressing functions are detected in various types of tumors [11,12]. One well-known *TP53* functional SNP is a genetic polymorphism Arg72Pro at codon 72 (rs1042522 C>G). This SNP has been well characterized, which can modify cancer susceptibility by affecting the function of p53 [13–16]. Numerous studies have indicated the association between the rs1042522 polymorphism and the risk of a variety of tumors, including breast cancer, prostate cancer, thyroid cancer, bladder cancer, hepatocellular carcinoma, cervical cancer, osteosarcoma, and Wilms tumor [17–25]. However, researches on the association between this SNP and neuroblastoma risk are very few. Therefore, we analyze the association in the eastern Chinese children population with 373 neuroblastoma patients and 762 healthy controls.

## Material and methods

### Study population

A total of 179 histopathologically confirmed neuroblastoma patients were enrolled from Anhui Provincial Children’s Hospital, Anhui, China. All the cases were newly diagnosed between October 29, 2008 and October 31, 2019. Moreover, 264 age- and gender-matched healthy volunteers, residing in the same region, were chosen as controls (Supplementary Table S1). Beside, an additional of 158 cases and 426 controls were enrolled from Children’s Hospital of Nanjing Medical University, Jiangsu, China) [26,27], and 36 cases and 72 controls enrolled from The Second Affiliated Hospital and Yuying Children’s Hospital of Wenzhou Medical University, Wenzhou area, China [28,29]. The Demographic characteristics for the 373 neuroblastoma cases and 762 controls were shown in Supplementary Table S2. Peripheral blood samples from all participants were taken to extract DNA prior to chemotherapy or radiation therapy. The present study was approved by the Ethics Committee of each hospital, while written informed consent was obtained from all the subjects’ parents or their legal guardians.

### Genotyping

The genotyping of *TP53* rs1042522 C>G was performed by Taqman real-time PCR method using a 7900 Sequence Detection System (Applied Biosystems, Foster City, CA). Taqman probes for allelic discrimination were purchased from ABI company [30–32].

### Statistical analysis

Statistical analysis was performed using the SAS software (version 9.1; SAS Institute, Cary, NC). The deviation from Hardy–Weinberg equilibrium was tested for the *TP53* rs1042522 C>G polymorphism in control subjects by a goodness-of-fit χ^2^ test. The association between *TP53* rs1042522 C>G and neuroblastoma susceptibility was estimated by odd ratios (ORs) and 95% confidence intervals (CIs) using an unconditional logistic regression adjusted by age and gender. All tests were two-sided, and *P* value less than 0.05 were considered significant.

## Results

### Clinical characteristics

The distributions of demographic characteristics of all participants are presented in Supplementary Table S2. Controls were frequency-matched to cases by age and gender. Mean age of cases and controls were 30.74 ± 29.08 and 33.04 ± 30.30 months old (*P*=0.770), respectively. Moreover, there was no significant difference in gender distribution between cases and controls (*P*=0.971). Additionally, 94 tumors (25.20%) located in adrenal glands, 145 (38.87%) in retroperitoneal, 110 (29.49%) in mediastinum, and 24 (6.43%) in other regions.

### Genotypic distribution of *TP53* gene rs1042522 C>G polymorphism

*TP53* gene rs1042522 C>G genotypes frequencies for neuroblastoma patients and controls are listed in Table 1. The genotypes for controls were consistent with Hardy–Weinberg equilibrium (*P*=0.241). The genotypic distribution of *TP53* gene rs1042522 C>G was as follows: 32.17% (CC), 49.33% (CG), and 18.50% (GG) in cases, and 30.58% (CC), 51.31% (CG), and 18.11% (GG) in controls. No significant difference were observed between cases and controls (CG vs. CC: adjusted OR = 0.92, 95% CI = 0.70–1.22, *P*=0.567; GG vs. CC: adjusted OR = 0.99, 95% CI = 0.69–1.42, *P*=0.947; CG/GG vs. CC: adjusted OR = 0.94, 95% CI = 0.72–1.23, *P*=0.639; or GG vs. CC/CG: adjusted OR = 1.04, 95% CI = 0.75–1.43, *P*=0.814), even adjusted for age and gender.

**Table 1:** Comparison between neuroblastoma patients and controls regarding *TP53* gene rs1042522 C>G genotypes frequencies in eastern Chinese children

Genotype	Cases (*N*=373)	Controls (*N*=762)	*P*^a^	Crude OR (95% CI)	*P*	Adjusted OR (95% CI)^b^	*P*^b^
rs1042522 C>G (HWE = 0.241)							
CC	120 (32.17)	233 (30.58)		1.00		1.00	
CG	184 (49.33)	391 (51.31)		0.91 (0.69–1.21)	0.530	0.92 (0.70–1.22)	0.567
GG	69 (18.50)	138 (18.11)		0.97 (0.68–1.40)	0.873	0.99 (0.69–1.42)	0.947
Additive			0.782	0.98 (0.82–1.17)	0.782	0.98 (0.82–1.18)	0.855
Dominant	253 (67.83)	529 (69.42)	0.586	0.93 (0.71–1.21)	0.586	0.94 (0.72–1.23)	0.639
Recessive	304 (81.50)	624 (81.89)	0.874	1.03 (0.75–1.41)	0.873	1.04 (0.75–1.43)	0.814

Abbreviations: CI, confidence interval, HWE, Hardy–Weinberg equilibrium; OR, odds ratio.

a χ^2^ test for genotype distributions between neuroblastoma patients and controls.

b Adjusted for age and gender.

### Stratification analysis

Stratification analysis by age, gender, and tumor sites were further performed to explore the association between rs1042522 C>G polymorphism and susceptibility to neuroblastoma. As shown in Table 2, no significant association was found.

**Table 2:** *TP53* gene rs1042522 C>G polymorphism and neuroblastoma susceptibility stratified by demographic characteristics in eastern Chinese children

Variables	Cases/Controls		Crude OR	*P*	Adjusted OR^a^	*P*^a^
	CC	CG/GG	(95% CI)		(95% CI)	
Age, month						
≤18	53/115	110/225	1.06 (0.71–1.58)	0.772	1.06 (0.71–1.57)	0.786
>18	67/118	143/304	0.83 (0.58–1.19)	0.305	0.83 (0.58–1.19)	0.311
Gender						
Females	46/105	120/235	1.17 (0.77–1.76)	0.464	1.21 (0.80–1.83)	0.375
Males	74/128	133/294	0.78 (0.55–1.11)	0.172	0.77 (0.54–1.10)	0.154
Sites of origin						
Adrenal gland	36/233	58/529	0.71 (0.46–1.11)	0.129	0.72 (0.46–1.12)	0.148
Retroperitoneal	46/233	99/529	0.95 (0.65–1.39)	0.784	0.95 (0.65–1.40)	0.801
Mediastinum	34/233	76/529	0.99 (0.64–1.52)	0.944	1.00 (0.65–1.54)	0.985
Others	4/233	20/529	2.20 (0.74–6.51)	0.154	2.37 (0.80–7.04)	0.121

Abbreviations: CI, confidence interval; OR, odds ratio.

a Adjusted for age and gender, omitting the corresponding stratification factor.

## Discussion

*TP53* is an important tumor suppressor gene, which is located on the short arm of chromosome 17; p53 protein plays a critical role in regulating cell growth, differentiation, and apoptosis [15]. The disruption or abnormally low transcription of *TP53* gene can impair the tumor suppressor function of the p53 signaling pathway, thereby promoting tumorigenesis and malignant progression [33]. The polymorphic locus rs1042522 C>G is located in exon 4 of the *TP53* gene, which leads to the conversion of arginine (Arg) to proline (Pro) at the 72nd codon [13]. Several lines of evidence has shown that this SNP is able to affect the function of p53, thereby modifying cancer susceptibility [13–16]. p53 is a transcription factor. It was revealed that the p53Arg and p53Pro displayed differential ability to transcriptionally activate target genes to trigger apoptosis, and to inhibit malignant transformation of cells [15].

Although the *TP53* gene rs1042522 C>G has been broadly studied for its role in cancer susceptibility [17–23], it should be noted that there are very few publications on the association between this SNP and neuroblastoma in childhood. In the present study, we aimed to explore the association between the *TP53* gene rs1042522 C>G and neuroblastoma in an eastern Chinese population. We genotyped this SNP in 373 neuroblastoma cases and 762 controls, and found that rs1042522 C>G might be not significantly associated with neuroblastoma susceptibility. In addition, stratification analysis by age, gender, and tumor sites suggested that CC genotype appears to have pathogenic effects in subjects not older than 18 months, but these results did not reach statistical significance.

He et al. previously investigated the association between the *TP53* gene rs1042522 C>G polymorphism and susceptibility to neuroblastoma in southern Chinese children [34]. With 256 patients and 531 controls, they reported no significant findings in main analysis, but significant association were detected in stratified analysis [34]. In Europe, Cattelani et al. [35] found that *TP53* gene rs1042522 C>G has no significant effect on the risk of neuroblastoma with 286 neuroblastoma patients and 288 healthy controls, but may be an indicator of poor prognosis. Taken together, it suggested that the rs1042522 C>G polymorphism may have a low-penetrant effect on the neuroblastoma risk. Large, or multi-center studies because of low incidence of the disease, are encouraged to dissect the effect of this SNP on neuroblastoma risk.

Several limitations should be noted. First, neuroblastoma is a multifactorial disease caused by the interaction between environmental factors and genetic background. Therefore, environmental exposures should have been also considered if data were available. Second, in the present study, only rs1042522 C>G in the *TP53* gene was investigated. Many other cancer susceptibility SNPs in the *TP53*, such as rs78378222 and rs35850753 [36], should be also investigated in the neuroblastoma.

By analyzing the association between *TP53* gene rs1042522 C>G and the risk of neuroblastoma in eastern Chinese children, we found that *TP53* gene rs1042522 C>G has no significant effect on susceptibility to neuroblastoma. However, due to the limitations of the sample size and observational indicator in the present study, multi-center investigations and analysis of other functional SNPs by whole-genome sequencing are required in the future to further validate our research result.

## Supplementary Material

Supplementary Tables S1-S2

## Abbreviations

CI: confidence interval
GWAS: genome-wide association study
HWE: Hardy–Weinberg equilibrium
OR: odds ratio
SNP: single-nucleotide polymorphism
