# Supplementary material for: TP53 Arg72Pro polymorphism and neuroblastoma susceptibility in eastern Chinese children: a three-center case–control study
Source: Biosci Rep. 2020 May 22;40(5):BSR20200854. doi: 10.1042/BSR20200854 (PMC7244897; doi:10.1042/BSR20200854)
Supplement: Supplementary Tables S1-S2 [file BSR-2020-0854_supp.pdf]

Supplemental Table 1. Demographic characteristics for neuroblastoma patients and controls from Anhui province

Variables	Cases (N=179)		Controls (N=264)		*P* [a]
	No.	%	No.	%	
Age range, month	0.001-132		0.001-96		0.173
Mean ± SD	29.94 ± 27.15		29.67 ± 27.52		
≤18	73	40.78	125	47.35	
>18	106	59.22	139	52.65	
Gender					0.563
Female	71	39.66	112	42.42	
Male	108	60.34	152	57.58	
Clinical stages					
I	61	34.08	/	/	
II	54	30.17	/	/	
III	28	15.64	/	/	
IV	29	16.20	/	/	
4s	7	3.91	/	/	
Sites of origin					
Adrenal gland	69	38.55	/	/	
Retroperitoneal region	60	33.52	/	/	
Mediastinum	37	20.67	/	/	
Other region	13	7.26	/	/	

SD, standard deviation.

[a] Two-sided χ^2 test for distributions between neuroblastoma patients and controls.

Supplemental Table 2. Demographic characteristics of neuroblastoma cases and cancer-free controls in eastern Chinese children

Variables	Cases (N=373)		Controls (N=762)		*P* [a]
	No.	%	No.	%	
Age range, month	0.001-132.00		0.001-132.00		0.770
Mean ± SD	30.74 ± 29.08		33.04 ± 30.30		
≤18	163	43.70	340	44.62	
>18	210	56.30	422	55.38	
Gender					0.971
Female	166	44.50	340	44.62	
Male	207	55.50	422	55.38	
Sites of origin					
Adrenal gland	94	25.20			
Retroperitoneal region	145	38.87			
Mediastinum	110	29.49			
Other region	24	6.43			

SD, standard deviation.

[a] Two-sided χ^2 test between neuroblastoma patients and cancer-free controls.
